# Monitoring the Spatiotemporal Activities of miRNAs in Small Animal Models Using Molecular Imaging Modalities

**DOI:** 10.3390/ijms16034947

**Published:** 2015-03-04

**Authors:** Patrick Baril, Safia Ezzine, Chantal Pichon

**Affiliations:** Centre de Biophysique Moléculaire, CNRS UPR4301, Université d’Orléans, 45071 Orléans, France; E-Mails: safia.ezzine@cnrs-orleans.fr (S.E.); chantal.pichon@cnrs-orleans.fr (C.P.)

**Keywords:** microRNAs, molecular imaging, spatiotemporal regulation, RILES (RNAi-inducing Luciferase expression system)

## Abstract

MicroRNAs (miRNAs) are a class of small non-coding RNAs that regulate gene expression by binding mRNA targets via sequence complementary inducing translational repression and/or mRNA degradation. A current challenge in the field of miRNA biology is to understand the functionality of miRNAs under physiopathological conditions. Recent evidence indicates that miRNA expression is more complex than simple regulation at the transcriptional level. MiRNAs undergo complex post-transcriptional regulations such miRNA processing, editing, accumulation and re-cycling within P-bodies. They are dynamically regulated and have a well-orchestrated spatiotemporal localization pattern. Real-time and spatio-temporal analyses of miRNA expression are difficult to evaluate and often underestimated. Therefore, important information connecting miRNA expression and function can be lost. Conventional miRNA profiling methods such as Northern blot, real-time PCR, microarray, *in situ* hybridization and deep sequencing continue to contribute to our knowledge of miRNA biology. However, these methods can seldom shed light on the spatiotemporal organization and function of miRNAs in real-time. Non-invasive molecular imaging methods have the potential to address these issues and are thus attracting increasing attention. This paper reviews the state-of-the-art of methods used to detect miRNAs and discusses their contribution in the emerging field of miRNA biology and therapy.

## 1. Introduction

The central dogma of molecular biology that a gene is transcribed into messenger RNAs (mRNAs) which are in turn translated into proteins was refined by remarkable works in the early 1990s. The discovery by Ambros and colleagues [1] that the *lin-4* gene controls the development of *Caenorhabditis elegans* via non-coding RNA molecules of 21–61 nucleotides without protein production considerably challenged this concept. At the same time, Ruvkun and colleagues [2] demonstrated that the expression pattern of the *lin-4* gene was the opposite of the *lin-14* gene which was known to be temporally regulated during *C. elegans* development. Sequence analysis revealed that the 3'-untranslated region of LIN-14 mRNA has a sequence complementary to *lin-4*, suggesting that these two molecules could interact through a base pair mechanism. In early 2000, a second non-coding RNA, let-7, was also identified in *C. elegans*. Genetic ablation of the *let-7* gene caused the L4-to-adult transition phase to reiterate, whereas ablation of the *lin-4* gene caused reiteration of the early L1 phase and death of animals. It was further demonstrated that *let-7* encodes a non-coding 21-nucleotide RNA that was complementary to the 3' untranslated region of many heterochronic genes, such as *lin-14*, *lin-28*, *lin-41*, *lin-42* and *daf-12*. Reporter gene assays bearing the 3' untranslated region of *lin-14* in the 3'-UTR of the *Lac-Z* gene confirmed that these two RNA molecules interact to induce the gradual repression of *Lac-Z* expression in the late phase of embryonic development through a post-transcriptional regulation mechanism. Soon thereafter, several *let-7* homologs were identified in many vertebrate species including humans, leading to the hypothesis that this mechanism of gene regulation is conserved throughout evolution and might also be crucial for many other biological processes. In the meantime, in 1998, Fire, Mello and colleagues [3] administrated non-coding double-stranded RNA molecules in *C. elegans* and demonstrated that these synthetic molecules were more potent in silencing the expression of heterochronic genes than conventional single strand antisense molecules. Molecular mechanisms underlying this process were again identified at the post-transcriptional level through a base-paring mechanism leading to the degradation of mRNAs by bound silencing RNA molecules. The authors also reported that the expression of the targeted genes was silenced by injecting only a small amount of molecules, suggesting the existence of an endogenous catalytic or amplification component. Today, it is known that these small non-coding RNAs are in fact miRNAs and that they mediate their post-transcriptional repression in a Drosha and Dicer dependent manner [4]. The importance of these two endoribonucleases is demonstrated by the disruption of the gene *Dicer1* in mice that results in early embryonic lethality [5] and in the arrest of zebrafish development on day 10 [6].

Research in the field of non-coding RNA has been advancing remarkably fast and remains a focus of intense research. One such class of non-coding (nc)RNAs are gene-regulatory miRNAs, which are 17–25 nucleotides long and control gene expression at the post-transcriptional level by either facilitating the degradation of mature mRNA and/or inhibiting the translation machinery [4,7]. After more than 20 years of extensive research, it is thought that the human genome encodes for 1200 miRNA genes and that at least 60% of the transcriptome is under the control of miRNA. It is therefore not surprising that deregulation of miRNAs has also been associated with a number of diseases and that RNAi-based therapeutic agents are currently being assessed as promising novel therapeutic drugs [8,9].

**Figure 1:**
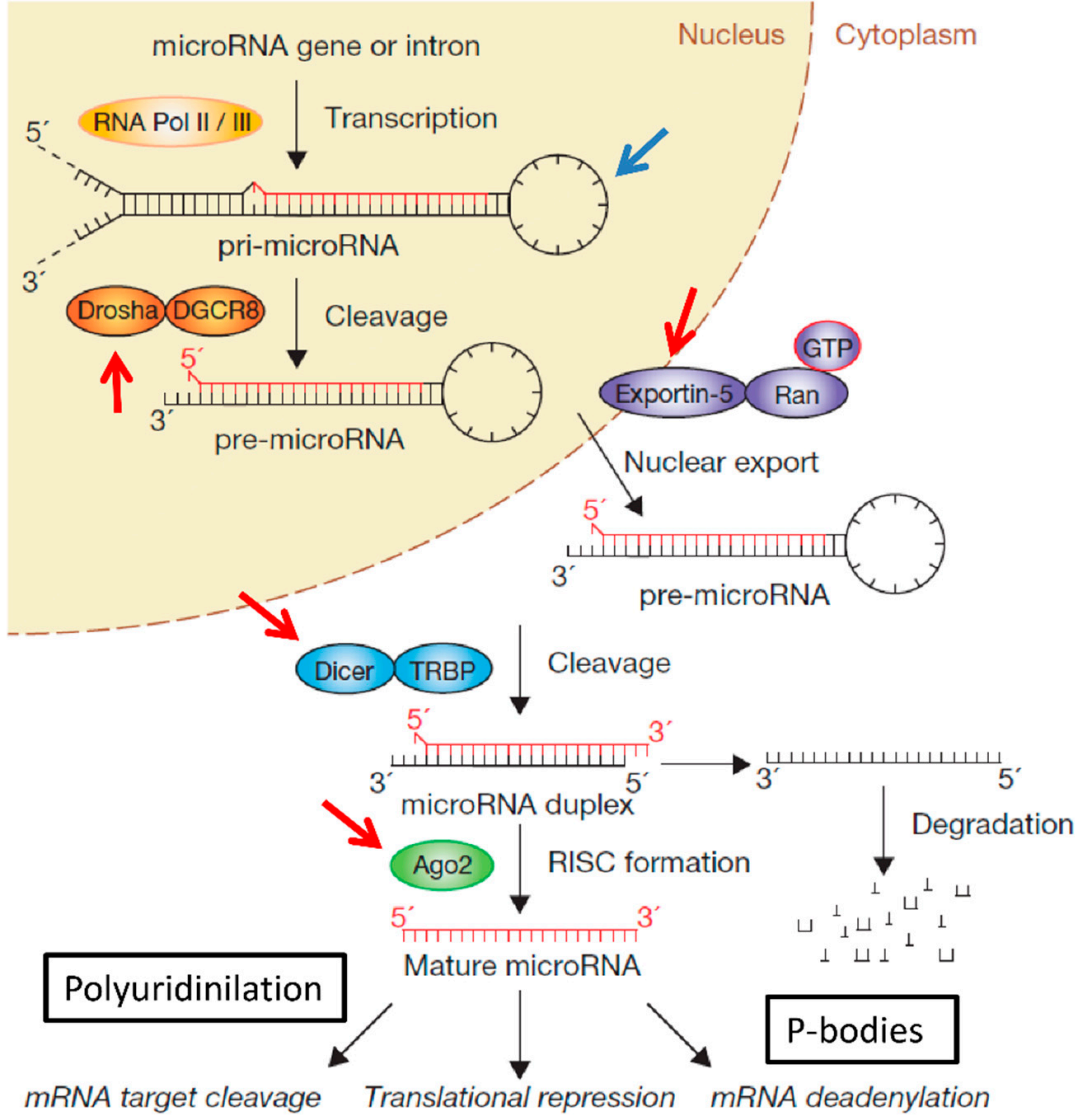
miRNA biogenesis and examples of post-transcriptional controls. MiRNAs are transcribed as pri-miRNAs by RNA polymerase II and processed by the endonuclease Drosha/DGCR8 to a shorter pre-miRNA hairpin structure. The pre-miRNA is exported to the cytoplasm by the Exportin-5–Ran-GTP and cleaved by DICER/TRBP complex to generate the mature length of the miRNA structure. One strand of the miRNA duplex, the guide strand, is then loaded with Argonaute (Ago2) proteins into the RNA-induced silencing complex (RISC), where it guides RISC to silence target mRNAs through mRNA cleavage or translational repression. Beyond this well described linear miRNA processing pathway, miRNA biogenesis is also subject to complex post-transcriptional controls. Some are indicated by the arrows in the figure. These include RNA editing of the pri-miRNA structure (blue arrow), regulation of miRNA biogenesis partners (red arrows), trafficking to P-bodies and polyuridylation for miRNA stability, cytosolic abundance and Dicer processing. Reprinted and adapted with permission from [4].

An intriguing aspect of miRNA biology is that a fraction of miRNA genes are located in intronic regions of both protein coding and non-coding genes [10]. Most miRNA genes are transcribed by polymerase II from individual transcriptional units or in frame with host genes either as a single transcript or as a polycistronic transcript composed of a cluster of several miRNAs [4,7,10]. A first endoribonuclease complex composed of Drosha and DGCR8 processes this primary miRNA (pri-miRNA) transcript in a RNA molecule of approximately 70 nucleotides, called precursor miRNA (pre-miRNA). The pre-miRNA is exported to the cytoplasm by exportin-5. Once in the cytosol, the pre-miRNA is processed by a second endoribonuclease complex consisting of DICER and TRBP which shortens the miRNA molecule to a 20–22 nucleotide-long duplex RNA molecule. This miRNA duplex is unwound into two separate single-strand RNA molecules. One of the strands, called the guide strand or mature miRNA, is incorporated into the RISC loading complex (RLC), composed of DICER, TRBP and Argonaute proteins bound to the mature miRNA. Once loaded in the RLC, miRNA interacts with its mRNA target sequences through a complementary base paring mechanism. The second strand, called the passenger strand (miR* strand), is degraded by an unknown or poorly described mechanism. Two different post-transcriptional mechanisms can take place (Figure 1) according to the degree of complementary sequence between the miRNA and mRNA. If there is perfect sequence match (100% homology), the mRNA is cleaved and degraded. On the other hand, if the sequence match is imperfect (less than 100% homology), the translational machinery is blocked in the initiation or elongation steps and/or the mRNA is degraded by deadenylation and decapping [8].

In addition to this well-described biogenesis pathway of miRNA expression, miRNAs also undergo complex post-transcriptional regulations which impact on their production and function [11]. As an example is the RNA editing process. In mammals, RNA editing is catalysed by two adenosine deaminases, ADAR and ADARB1, which recognize double-stranded RNA and convert adenosine (A) into inosine (I) [12]. The double-stranded stem-loop structure of the precursor form of miRNA is a natural substrate for ADAR enzymes. The editing causes a slight change in the miRNA molecule sequence resulting in their complementary bound sequence being redirected to novel mRNA targets. This miRNA editing process is critical for numerous physiological processes [13,14] and also for pathological processes such as cancer [15,16]. It has been reported that several types of tumours have altered levels of RNA editing [15]. Recently, the accumulation of miRNA editing has been found to be positively correlated with the extent of invasive tumour spread in human patients with glioblastoma tumours [17]. Although it is unclear whether these alterations are the cause or the consequence of cancer development, it has been hypothesised that cancer cells can edit miRNAs to modulate their uptake through the RISC machinery to change their tumoral phenotype [18,19]. Another example of post-transcriptional control of miRNA activity is the altered trafficking of miRNA in specific cytoplasmic granules such as P-bodies. P-bodies are cytoplasmic foci involved in the post-transcriptional regulation of eukaryotic genes by controlling mRNA turnover and degradation [20]. The evidence that most RNAi machinery proteins (mainly DGCR8 and Ago) are localized in P-bodies with miRNAs and their respective mRNAs suggests that P-bodies might play a role in the control of gene expression exerted by miRNAs [21]. It was suggested that miRNAs bound to target mRNAs could be redirected to P-bodies either to exert their catalytic function or to be stored temporally [22,23]. For example, the cationic amino acid transporter-1 (CAT-1) mRNA is a target of miRNA-122 in hepatoma HuH 7 cells which are localized in P-bodies with miRNA-122 under physiologic conditions. MiRNA-122/CAT-1 complexes are released from P-bodies under stress conditions and negatively repress the expression of CAT-1 mRNA as evidenced by quantitative PCR (qRT-PCR) [24]. An interplay between miRNAs and P-bodies has recently been reported in the context of the hepatitis C virus replication. The P-body protein LSm1 was found to contribute to the stimulation of hepatitis C virus translation, through a miRNA-122-dependent mechanism [25]. However, it should be noted that the exact involvement of P-bodies in regulating miRNA-mediated gene expression remains under debate. More studies are required to clarify this point. The stability of miRNA is also subject to complex post-transcriptional control. For instance, the addition of non-coding nucleotides at the 3'-end of mature and pre-miRNA affects miRNA stability, its cytosolic abundance and its processing by Dicer. This process is known as uridylation or adenylation of miRNAs [4]. Addition of several uridine nucleotides (polyuridylation) to the 3'-end of miRNA-26 abrogates IL-6 repression [26] whereas, Zcchc11 (a RNA uridyltransferase) deficiency diminished the lengths and terminal uridine frequencies of several mature miRNAs and modulated neonatal IGF-1 expression growth and survival [27]. Precursor forms of some miRNAs can also be polyuridylated. For example, addition of uridine nucleotides to pre-let-7 miRNA prevents Dicer processing and induces precursor miRNA degradation [28,29]. As for polyuridylation, precursor forms of miRNAs can also be polyadenylated. Lastly Cai *et al.* [30] demonstrated that at least nine human pre-miRNAs are capped and polyadenylated suggesting that both stability and activity of this non-coding RNA transcript might be regulated as described for coding RNA transcripts [30]. Furthermore, a single addition of adenine nucleotide (adenylation) to the 3'-end of mature miRNAs appears to modulate miRNAs activity and stability. For example, adenylation of the 3'-end of miRNA-122 prevents trimming and protects miRNA against exonucleolytic degradation in liver cells through a process involving the noncanonical poly(A) polymerase (PAPs) GLD-2 [31,32]. Many other post-transcriptional controls of miRNA biogenesis and activity have been reported [4]. Some examples include the deregulation of exportin-5 [33], DGCR8 [34] and Ago-2 [35] expression and loss of DICER activity in cancer cells [36].

Overall, these findings indicate that miRNA biology is complex, highly dynamic and subject to tight transcriptional and post-transcriptional control. All of these events contribute to the spatial and temporal patterns of miRNA expression which in turn fine-tune the expression of gene networks. The underestimation of these features results in the loss of important information linking miRNA expression to cell function. It is therefore crucial to know the repertoire of miRNAs and the change in their expression in response to cellular environments to better understand their biological functions. In the past 10 years, a plethora of detection methods has been developed to monitor miRNA expression. However, monitoring of miRNAs is difficult due to their intrinsic physicochemical characteristics, such as their small size, low abundance and high degree of sequence similarity between miRNA family members for instance. Current monitoring methods including Northern-blot, real-time PCR, microarray, *in situ* hybridization and deep sequencing have largely contributed to our understanding of miRNA biology and will undoubtedly continue to provide important insight into their biological roles and regulation. However, these methods can seldom shed light on the spatiotemporal organization and function of miRNAs in real-time. Indeed, all of these methods, with the exception of *in situ* hybridization, require cell lysis to access the miRNA population. Thus, they can hardly assess the spatial and temporal patterns of miRNA expression as well as the functionality of miRNA. As mentioned above, the detection of mature miRNAs does not automatically mean that they are biologically active, e.g., processed by the RLC complex. Recently, a high-throughput assay, called Sensor-seq, has been developed to assess simultaneously the activity and function of a hundred miRNAs using decoy or sponge miRNA libraries [37]. Using this approach, it has been demonstrated that only the most abundant miRNAs within a cell population mediate significant target suppression. Surprisingly, only 60% of the miRNA population was able to repress expression of target mRNA, suggesting that the functional “miRNome” in the cell is smaller than originally thought.

In this context, non-invasive molecular imaging methods have the potential to overcome some of these hurdles and to provide alternative ways to study miRNA expression in physiological and pathophysiological animal models. These methods performed on live-anesthetized animals can be repeated overtime allowing the longitudinal analysis of miRNA expression and the study of spatial restriction pattern of miRNA. Moreover, some of these methods monitor the active form of miRNAs processed by the RNA silencing machinery.

In this review, we provide a comprehensive state-of-the-art of methods used to monitor miRNA expression under physio(patho)logical conditions (Table 1). The advantages and limitations of these monitoring systems and their contribution to the field of miRNA research are discussed. These monitoring methods can be subtyped into three classes: conventional, synthetic and biological miRNA probes. The first corresponds to the conventional invasive methods used to detect miRNA. They include Northern-blot, PCR-based approaches, microarray analysis, the deep sequencing method and *in situ* hybridization. The second concerns synthetic probes that rely on oligoribonucleotide sequences which are complementary to a mature miRNA. These synthetic molecules are linked to fluorophores which emit light on binding to their miRNA target. The third involves miRNA probes which use fluorescent or bioluminescent reporter genes expressed directly under the control of a target miRNA. According to the design of the imaging system, an increase or reduction in light emission in cells is generated and can be detected using conventional optical imaging methods.

## 2. Conventional Methods for miRNA Detection: PCR, Northern-Blot, Deep Sequencing and *in Situ* Hybridisation (ISH)

The unique physicochemical properties of miRNAs greatly hinder their detection [38,39,40,41]. Probe design is limited due to the very short and important miRNA sequence homologies. Thus, optimal hybridization temperatures are required for a probe to bind specifically to a miRNA sequence without generating imperfect hybridization or biased interpretations. Furthermore, it is estimated that the fraction of the miRNA pool in cells represents on average 0.01% of the total RNA; therefore, amplification procedures are required to monitor miRNAs faintly expressed in cells. Consequently, these procedures could create additional bias during the different steps of the experimental protocol. Finally, as some miRNA sequences differ in only one or two nucleotides, it is a real challenge to discriminate miRNA family members, for instance. Intensive efforts are ongoing to develop novel miRNA monitoring methods to address these problems. Historically, Northern-blot, *in situ* hybridization, microarray and PCR based approaches were the first implanted methods to detect the expression of miRNA. More recently, the deep sequencing method has extended the repertoire of these so-called conventional and invasive methods. All of these methods display a common first step. The cell or tissue source of miRNA expression has first to be lysed and/or fixed before being hybridized with a specific miRNA probe.

Northern-blot was the first detection method developed to detect miRNAs expression in samples. This method contributed considerably to the discovery of miRNA in early 1990 and has notably highlighted the crucial temporal regulation aspect of miRNA during embryonic development [42]. The main advantage of this method is that the probe used in Northern-blot can determine the size of different forms of miRNA, from the long primary and precursor forms to the short mature form. This procedure is sensitive enough to detect the expression of a given miRNA from a pool of total RNA fraction without requiring an amplification step. Experimental procedures have demonstrated that as few as 3-bp mismatches between miRNA molecules is sufficient to confer probe specificity and accurate detection of miRNA. Higher binding affinity and specificity between probes and miRNA can be improved further by incorporating chemical modifications in the oligonucleotides probes. The 2'-*O* methyl modification of nucleotides is known to increase stability and also protects against nucleases, while locked nucleic acid (LNA) modification of nucleotides increases the melting temperature (Tm) providing more stringent conditions [43]. Overall, Northern-blot is still considered as the best qualitative method to monitor several forms of miRNA in complex samples. A clear advantage of this approach is that no amplification step is required. For each miRNA of interest it is possible to optimize the Tm to improve sensitivity and reduce non-specific hybridization. However, this method is time-consuming (two to three days), requires a large amount of starting RNA material (minimum of 10 µg) and can hardly assess the functionality of miRNAs (Table 1).

Microarray analysis provides a high-throughput analysis of multiple miRNAs expressed by the same cell population. This method is widely used to screen for novel diagnostic markers for clinical use [44]. The miRNA array consists of 4000 probes printed in duplicate to monitor simultaneously more than 1500 mature miRNAs and corresponding precursors in 10 samples, processed at the same time in a single experiment. The drawback of the multiscreen system of miRNA expression is that a unique temperature is required to hybridize the miRNA populations of all spotted probes on microarray chips. As outlined above, according to the GC content and sequence of miRNA molecules, the Tm to hybridize miRNAs to the probes can vary between 45 and 74 °C. The arbitrary choice of a given Tm temperature can therefore generate imperfect and/or non-specific hybridization. This procedure is more suitable for determining the relative change in miRNA expression between two states, for example, non-treated *versus* treated samples and/or healthy *versus* diseased samples. It is possible to adapt the length of the spotted probes according to the physicochemical characteristics of each targeted miRNA and to incorporate LNA and 2'-*O* methyl modifications on the spotted oligonucleotide probes to improve sensitivity and specificity. Nowadays, microarrays incorporate these modifications and provide normalized Tm for comparable binding of the miRNAs to the probes [45]. Overall, this approach is qualitative and also quantitative, although the quantitative aspect of the microarray analysis generates some debate. As for Northern-blot, this approach is not appropriate to identify novel miRNA sequences and can hardly distinguish miRNA family members. Finally, microarray procedures require several steps to extract, purify, amplify and label the miRNA population before it can be hybridized on the arrays.

The quantitative PCR of miRNA is of interest. This method relies on a reverse transcription step to convert miRNAs into cDNAs, followed by a real time PCR step performed in the presence of a fluorescent probe (Taqman™ or Sybergreen dyes). Due to the small size of miRNA, a chemical adapter is first linked to the miRNA population to generate a PCR product (amplicon) of between 150 and 250 base pairs for accurate quantification. There are several methods to reverse transcribe a miRNA into a longer cDNA molecule. These include the stem-loop primer and the poly (A) tail addition methods [46,47]. All have advantages and drawbacks, but to date the most widely used method is the poly (A) tail addition method. The elongation of the miRNA sequence is carried out by a polyadenylation reaction of the 3'-ends of the miRNA population using *E. coli* poly(A) polymerase. A universal RT primer consisting of the 3'-end a oligo(dT) sequence is then used to bind to the poly (A) tailed miRNA and prime the reverse transcription. The PCR step is then performed with forward primers specific to the miRNA sequence and reverse, universal primer specific to the poly(A) tail [48,49]. This approach is particularly useful to detect several miRNAs from the same cell population. This monitoring method is fast, affordable and efficient, but depends on the possibility to generate a PCR amplicon without ambiguity. Although chemical optimised primers can be used (2'-*O*-Methyl and/or LNA chemistry primers), some miRNA sequences are difficult to amplify without generating non-specific amplicon products. In addition, this method can hardly discriminate miRNAs which differ in as few as three nucleotides, making the detection of miRNA family members challenging. Overall, this method is considered as an excellent first line approach to monitor expression of miRNA from different samples and can be performed easily in any laboratory familiar with quantitative PCR methods.

The deep sequencing approach has unique features which in the near future will generate novel information about miRNA expression, sequence and regulation [50]. The principle relies on preparing a miRNA library from a pool of purified mRNAs according to their size, to isolate pri-miRNA, pre-miRNA and mature forms of miRNAs. A ligation step is then performed to add an adaptor molecule to the 5'- and 3'- ends of the miRNA molecules. This adaptor is used as a matrix to prime a reverse transcription step. The RT products then undergo massive parallel sequencing which generates millions of small RNA sequence reads. This is probably the most important feature of deep sequencing [51]. It is a valuable method to determine novel miRNA sequences without ambiguity and to discriminate several miRNA members differing by only one nucleotide. To date, it is the only experimental detection method capable of achieving this at an unreached level of specificity and sensitivity. However, deep sequencing has several disadvantages, in particular its high cost, infrastructure requirements, expertise, and the generation of massive bioinformatics data that require complex algorithm computational methods for their analysis. Deep sequencing generates up to 3 Gbp of sequence reads on each run. This approach is now preferred to the microarray method to determine precise miRNA signatures for diagnosis of human diseases from tissue, blood and urine samples.

The *in situ* hybridization (ISH) method provides a unique opportunity to visualize the spatial expression pattern of miRNA in fixed tissues [52]. This approach has enabled assessment of the spatial restriction pattern of mammalian miRNAs in several vertebrate species including in human tissues [53]. In addition, this method provides single cell resolution enabling changes in subcellular distribution of miRNAs in organs/structures such as P-bodies to be monitored. The ISH procedure uses an oligonucleotide probe which is complementary to a miRNA sequence. The probe can carry a fluorophore for fluorescence-based analysis, an enzyme for colorimetric-based analysis or a radioactive probe for autoradiography-based analysis. Whilst ISH of mRNA is considered as a routine procedure, ISH of miRNA is challenging because of the small size and the low abundance of miRNA. To circumvent these hurdles, several methods have been developed to increase the sensitivity of the ISH procedure. Nowadays, ISH miRNA probes almost systematically contain LNA-nucleotides to strengthen their affinity and to stabilise the miRNA/mRNA complex during the washing steps performed under stringent conditions [54]. In brief, the ISH system is a reliable method to visualize the spatial location of miRNAs in cells and tissues, but requires extensive serial tissue section and optimized, time-costly procedures to generate a good signal-to-background ratio.

Despite their advantages, these methods can hardly assess the dynamics of miRNA expression overtime. Consequently, crucial information about miRNA regulation and functions is missing. This important issue can be overcome thanks to the development of new alternative approaches compatible with *in vivo* monitoring of miRNA under physiological conditions.

## 3. Molecular Imaging Using Synthetic Fluorophore-Labelled Probes

Synthetic probes used to monitor miRNA expression are based on the principle that a chemical oligoribonucleotide probe designed to have a complementary sequence to a miRNA, can specifically bind to it, through a base pair mechanism. If this synthetic probe is coupled to a sensitive fluorophore, it is possible to detect the expression of a miRNA target using fluorescence-based equipment.

Molecular beacons (MBs) are the most sophisticated chemical probes for miRNA. MBs are oligonucleotide probes of 25–30 nucleotides which spontaneously form a self-assembled stem-loop RNA structure. The probe is designed in a way that the loop sequence is perfectly complementary to the miRNA of interest, while the stem is grafted with a fluorochrome at its 5'-end and a quencher at its 3'-end. The native stem loop RNA structure imposes a closed proximal distance between the fluorophore and the quencher resulting in no fluorescence being emitted by the fluorochrome upon light excitation. Under this configuration, the MB is switched “OFF” and no signal is detected inside the cells. In contrast, when the miRNA of interest is expressed, it binds to the loop structure of the MB and induces its linearization. As a consequence, the fluorophore is physically separated from the quencher and can emit fluorescence on light activation [55,56].

This fluorescent-based monitoring system is sensitive, specific and a fast procedure capable of distinguishing mature and pre-miRNAs. Baker *et al.*, 2012 [57] demonstrated that a MB designed to detect miRNA-21 can readily distinguish mature and pre-miRNAs forms of this miRNA and reliably quantify miRNA expression *in vitro*. The same authors found that MBs with DNA, RNA and combined locked nucleic acid (LNA)–DNA backbones can detect miRNA concentrations as low as 1 nM with high specificity. Furthermore, MBs have the potential to distinguish miRNAs with close sequence homology when appropriate design and optimized hybridization temperatures are used [57]. A major advantage of this system is that each MB can accommodate several types of fluorophores with different emission peaks, enabling the monitoring of several miRNAs at the same time and in the same cells [58]. This system has been employed to monitor simultaneously the biogenesis of miRNA-206 and miRNA-26a during the differentiation process of C2C12 myoblast cells in myotubes *in vitro* [59]. A green-emitting MB probe (FAM, 6-carboxyfluorescein) was used to monitor the expression of miRNA-206, while a second MB probe with red emission (Texas red) was used to monitor miRNA-26a expression. *In vitro* and to a lesser degree *in vivo* fluorescence analyses revealed a gradual increase in fluorescence signals in C2C12 cells undergoing myotube formation due to the interaction of miRNA-206 and -26a with their respective MBs. Another benefit of this molecular imaging system is that it can be used to monitor the dynamic changes of miRNAs, over short periods of time (from minutes to hours).

This method has been successfully employed to monitor epithelial mesenchymal transition (EMT) of epithelial and breast cancer cell lines in real-time using magnetic MBs specific to miRNA-200a [60]. When these MBs were delivered to epithelial cells undergoing EMT upon treatment with 10 ng/mL of TGF-β1, the fluorescence of the probe was observed within 2 min 30 s, and increased in a time-dependent manner. Interestingly, the increase in fluorescence emission correlated directly with the degree of EMT attested by changes in cell morphology and loss of cell–cell adhesion. No toxicity or changes in endogenous miRNA-200a expression or transcription factors such as ZEB1, Snail or Slug mRNA were observed demonstrating that this imaging system interfered minimally with the cell biology. Recently, a MB probe composed of double-stranded locked nucleic acid (dsLNA) was used to detect the spatiotemporal localization of miRNAs during the epithelial migration [61]. Data generated using a model of wound healing *in vitro* revealed that miRNA-21.5 was enriched in the first 100 µm of the leading edge of the wound, where the cells migrated and proliferated. As for the work cited above [60], a dose- and time-dependent induction of miRNA-21.5 was detected in epithelial cells cultured in the presence of TGF-β suggesting that this miRNA could be functionally involved in the epithelial mesenchymal transition [61]. A similar strategy was also used to monitor miRNA-155 expression during the development of lung cancer cells in an animal model [62]. Data indicated that the emission of fluorescence was proportional to the dose of miRNA-155 transfected in tumour cells *in vitro*, while *in vivo* experiments performed with a fluorescence stereomicroscope demonstrated good sensitivity and specificity to the tumour cells. In this context, MBs might have applications in clinic, including tumour diagnosis. Indeed, this monitoring system is sensitive, faster and easier to perform than the qRT-PCR method and would provide the opportunity to screen for multiple miRNA tumour markers on the same clinical histology tissue section.

**Figure 2:**
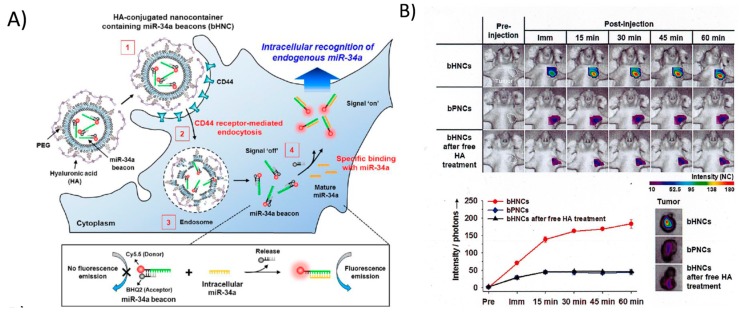
(**A**) Schematic illustration of theranostic miRNA-34a molecular beacon (MB) delivery system to target breast cancer cells *in vivo*. The system is based on hyaluronic acid coated-nanocontainers which bind to CD44 receptors of tumour cells (1) for efficient internalizationin endosomes (2); Next, the miRNA MB is released from the nanoparticles under acidic pH (3) and can bind to the miRNA-34a in the cytosol (4); Upon binding, the fluorophore Cy5.5 is no longer quenched by the BHQ2 quencher and can emit fluorescence upon excitation at the requested wavelength; and (**B**) *In vivo* and *ex vivo* imaging of miR-34a in an orthotopic breast cancer model. *Top panel.* *In vivo* optical fluorescence images of MDA-MB-231 tumour-bearing mice after intravenous injection of either miRNA-34a MBs (bHNCs), control nanocarriers without miRNA-34a MB (bPNCs) or mice treated with bHNCS *plus* free hyaluronic acid; *Lower left panel*. Total photon counts in tumour regions after administration of bHNCs, bPNCs, and bHNCs *plus* free HA treatment; *Lower right* *panel*: *Ex vivo* optical fluorescence images of tumours excised at 1 h post-injection of bHNCs, bPNCs, and bHNCs *plus* free HA treatment, respectively. Reprinted and adapted with permission from [65].

Like all nucleic acids, MBs are not easily taken up by cells and that has led to the development of different delivery systems. Moreover, the possibility to encapsulate small miRNA probes in nanoparticle delivery systems has stimulated the development of multifunctional imaging platforms to simultaneously detect and treat tumour cells *in vivo*. The first method was reported by Hwang *et al.* [63] who developed magnetic fluorescent nanoparticles composed of rhodamine-coated cobalt ferrite combined with a fluorescent miRNA-124a MB. These nanoparticles enabled the monitoring of miRNA-124a expression during neuronal differentiation using optical fluorescence imaging while the biodistribution of these nanoparticles were determined using magnetic resonance imaging (MRI). Enhanced fluorescence intensity was detected in cells undergoing neuronal differentiation *in vitro* and *in vivo.* This was reversed by the pre-treatment of cells with an inhibitor such as antagomiR-124a. In a follow-up study, Kim *et al.* [64] developed the first reported theranostic probe to detect and inhibit miRNA-221 expression during the development of cancer. This synthetic probe consists of an AS1411 aptamer and rhodamine-labelled miRNA-221 MB conjugated with a magnetic fluorescence nanoparticle used as a nanocarrier. The AS1411 aptamer was used to target the delivery of nanoparticles to the cancer cells and subsequently their cellular uptake by a cell-receptor internalization process. Once the nanoparticles reached the acidic environment of the endosomes, MBs were released from the nanoparticles and they became free to bind to the miRNA-221. As a consequence, they emit fluorescence on excitation of the rhodamine probe. Saturable sequestration of miRNA-221 by the miRNA-221 MBs was observed which in turn restored expression of tumour suppressor genes inducing significant antitumor therapeutic effects [64]. In the same year, a smart nanoprobe composed of pegylated nanocontainers consisting of Cy5-BHD2 labelled miRNA-34a MB and biodegradable hyaluronic acid was developed to detect and treat breast tumour cells in mice [65]. MiRNA-34a is a tumour-suppressor gene, down-regulated in several types of cancer cells and known to control the expression of several oncogenes involved in the cell cycle, differentiation, apoptosis and chemoresistance [66]. Due to its extensive antitumor effect, miRNA*-*34a replacement therapy is currently under evaluation in clinical trials [67]. Intravenous administration of the smart miRNA-34a MBs in tumour-bearing mice indicated that fluorophore-labelled nanoprobes were stable in mice, had a long blood circulation and did not create adverse events or toxicity in animals. Fluorescence optical imaging demonstrated that MBs loaded in these nanocontainers were efficiently delivered to metastatic tumour sites through a CD44 receptor-dependent endocytosis process, while healthy organs did not exhibit any fluorescence signal above the background value. It was further demonstrated that miRNA-34a MBs payloads delivered to the tumour were sufficient to induce apoptosis of tumour cells as demonstrated by the reduction in tumour volume and the detection of necrotic foci in tumour samples using histological analysis (Figure 2).

Despite promising results generated with fluorogenic miRNA MBs, this approach has some limitations. The main one is the low signal-to-background ratio due to *in vivo* autofluorescence and poor tissue penetration of excitation light [68]. Work is currently being carried out to address these issues with, for example, the development of near infrared fluorescence probes [69]. In this particular context, reporter genes encoding for fluorescent proteins or an enzyme enabling light emission from a substrate are more sensitive and have been fully validated [70]. Upon gene transfer, even at low efficiency, the amount of reporter protein produced by cells is superior to which can be achieved upon delivery of synthetic exogenous probes. The sensitivity of optical signals generated with reporter genes is therefore higher than that generated by chemical fluorophores [71].

## 4. Molecular Imaging Using Biological Probes: Applications in Basic Research and RNAi-Based-Therapy Including Gene and Cell Therapy

### 4.1. Negative Read out miRNA Monitoring Systems

There are two different types of read-out reporter gene systems to monitor miRNA expression. The first consists of a system based on extinction of optical signals generated by reporter genes [72]. This system is referred to as the “negative read-out system” or “miR-OFF monitoring system”. It relies on sub-cloning four perfect complementary block sequences of a given miRNA in the 3'-UTR region of the reporter gene driven by a ubiquitous promoter. This sequence is usually referred to as miRNA target sequence, miR T. When the target miRNA is expressed, it can bind to the miR T sequence inducing cleavage of the mRNA reporter through activation of the RNAi machinery. As a result, no reporter gene expression is produced, resulting in loss of light emission in transfected cells [73]. This strategy was first used to monitor the *in vivo* activity of miRNA- 142-3p expression in hematopoietic cells [74] and the expression of miRNA-133 during the differentiation program of C2C12 myoblasts into myotube *in vitro* [75]. Both systems are based on lentiviral and retroviral expression vectors for efficient expression of the reporter miR T probes in cells. With the first approach, it was found that the expression of miRNA-142-3p effectively suppressed the expression of the green fluorescent protein (GFP) reporter gene in transduced hematopoietic cell lineages, whereas GFP expression was maintained *in vitro* in non-hematopeotic cells such as 293T kidney cells, HUH7 hepatocarcinoma cells, and *in vivo* in the stroma cells of the liver, lungs, spleen and thymus [74]. It has further been demonstrated that this approach can be used to restrict expression of therapeutic transgenes in targeted cells according to the expression pattern of miRNA in these cells. This approach, known as the “de-targeting approach”, aborts the expression of therapeutic genes in antigen presenting cells, preventing the development of the immune response against the transgene, overcoming one of the most current issues in gene therapy [76]. Similarly, a two-colour retroviral vector system has been developed to monitor the dynamic of miRNA expression in living cells. In this imaging system, the GFP reporter gene was fused with four complementary bound sequences of the miRNA-133 to monitor its activity in myoblast cells, whereas the red fluorescent reporter gene (RFP) driven by an independent ubiquitous promoter was used to normalize the expression. The transduction of C2C12 myoblast cells resulted in the gradual loss of GFP expression in formed myotubes which was correlated with a gradual increase in miRNA-133 expression in these cells. By contrast, the expression of GFP was maintained as expression of miRNA-133 was not significantly expressed in undifferentiated C2C12 cells. Real-time imaging of GFP expression in living cells over seven days in culture provided precise fine-tune expression of miRNA-133 during the course of skeletal muscle differentiation *in vitro* [75]. These data revealed that miRNA expression is induced from the intermediate to late phase of the differentiation program of C2C12 cells, thus providing a better understanding of the interconnection between the MyoD transcription factor, miRNA-133 expression and skeletal muscle differentiation. More recently, a dual optical imaging system, composed of themiR-OFF gene reporter system and miR-ON fluorogenic MBs was also developed [77] to visualize induction of miRNA-1 during the myogenic differentiation of C2C12 cells. This dual optical miRNA imaging systems enabled to visualization of miRNA-1 biogenesis during the differentiation of C2C12 into myotubes both *in vitro* and *in vivo* using luciferase- and fluorescence-based imaging modalities. This approach provides complementary imaging information about miRNA biogenesis, thus overcoming the shortcomings of each miRNA imaging system taken individually [77].

Similar “miR-OFF systems” have been employed to monitor the biogenesis of several miRNAs and pri-miRNAs. In one report a dual-luciferase expression system was developed to monitor simultaneously the activity of miRNA-23a promoter using the firefly luciferase (FLuc) as reporter gene and the production of the mature form of the miRNA-23a using the Gaussia luciferase (GLuc) reporter gene [78]. In the latter, the miR T sequence composed of three complementary block sequences of this miRNA was fused to the 3'-UTR region of the GLuc reporter gene. Transfection of these two constructs in P19 neural or 293 kidney cells followed by their engraftment in nude mice resulted in an increased detection of FLuc emission in animals which correlated with a decrease in GLuc signals due to the production of the mature form of miRNA-23a. Interestingly, the production rates of precursor miRNA-23a and mature miRNA-23a were found to be different in the P19 and 293 cells indicating that miRNA biogenesis is a complex and cell-type dependent process. Using the same strategy, the same team also monitored the expression pattern of the brain-specific primary miRNA-9 during neurogenesis *in vitro* and *in vivo* [79]. The miR-OFF construct was used to determine which form, at the 5'- and 3'-end of the precursor miRNA structure was the more stable strand when processed as a mature miRNA duplex [79]. To investigate this point, the two mature forms of miRNA-9 (guide, miRNA-9 and passenger miRNA-9*) were produced either from the 3'-end or from the 5'-end of pre-mIRNA-9 constructs transfected in P19 neuronal cells expressing either the miR-9 or miR-9* OFF system. Comparison of bioluminescence signals emitted in mice indicated that miRNA 9 was more efficient than miRNA-9* in repressing the expression of the reporter gene. This observation generated under physiological conditions confirms that the guide strand of miRNA is more thermostable than the passenger strand. In other methods, this loss of reporter gene expression has also been employed to monitor the efficacy of non-viral delivery systems to deliver synthetic miRNA-16 molecules to xenografted prostate cancer cells. Tumour cells were engineered to express the Renilla luciferase (RLuc) containing complementary block sequences of this miRNA in the 3'-UTR region of this reporter gene. Intravenous administration in mice of miRNA-16/atelocollagen nanoparticles reduced RLuc expression by 50%, demonstrating the efficiency of this delivery system to reach tumour cells *in vivo* and to deliver its payload functionally in the cytosol [80]. The injection of miRNA-16/atelocollagen complexes significantly inhibited the growth of prostate tumours grafted in mice by repressing expression of *CDK1* and *CDK2* genes which are involved in cell-cycle control and proliferation.

This miRNA-off signal system is nowadays widely used *in vitro* in functional studies to confirm the presence of potential miRNA binding sequences in the 3'-UTR or 5'-UTR of target mRNA. *In vivo*, these miR-OFF system strategies have been found valuable to confirm the presence of the target sequence of miRNA-221 in the 3'-UTR of homeobox B5 mRNA of xenografted papillary thyroid carcinoma cells [81] and the presence of the target sequence of miRNA-124a in the 5'-UTR region of an unclassified gene located on chromosome 14 (c14orf24) [82].

A variant of this system called dual-fluorescence FunREG (functional, integrated and quantitative method to measure posttranscriptional regulations) was also developed [83]. Recently, this FunREG system has been used to screen a library of 876 individual miRNAs to identify miRNAs involved in regulating the Glypican-3 (GPC3) mRNA in hepatocarcinoma cell lines. The results of this functional screening indicate that miRNA-96 and its paralog miRNA-1271 repressed GPC3 expression, whereas miRNA-129-1-3p, miRNA-1291 and miRNA-1303 induce its expression. Notably, miRNA-1271 was found to be down-regulated in human hepatocarcinoma tumour samples and inversely correlated with the expression of GPC3 mRNA [84].

Despite their wide use, these off-reporter systems work in a negative way, with the loss of emitted optical signals indicating the miRNA of interest is expressed in cells. Therefore, this “negative” imaging mode could be inappropriate, particularly when considering *in vivo* applications. The lack of detection signals in animals during the course of an *in vivo* molecular imaging procedure is detrimental as it may reflect either poor diffusion of the luciferin substrate, a change in tissue photon absorption, technical issues with the digital charge-coupled device (CDD) camera, nonspecific regulation of the reporter gene promoter and/or even cell death. Therefore, to overcome these limitations, positive read-out miRNA imaging methods have been developed.

### 4.2. Positive Read-out miRNA Monitoring Systems

**Figure 3:**
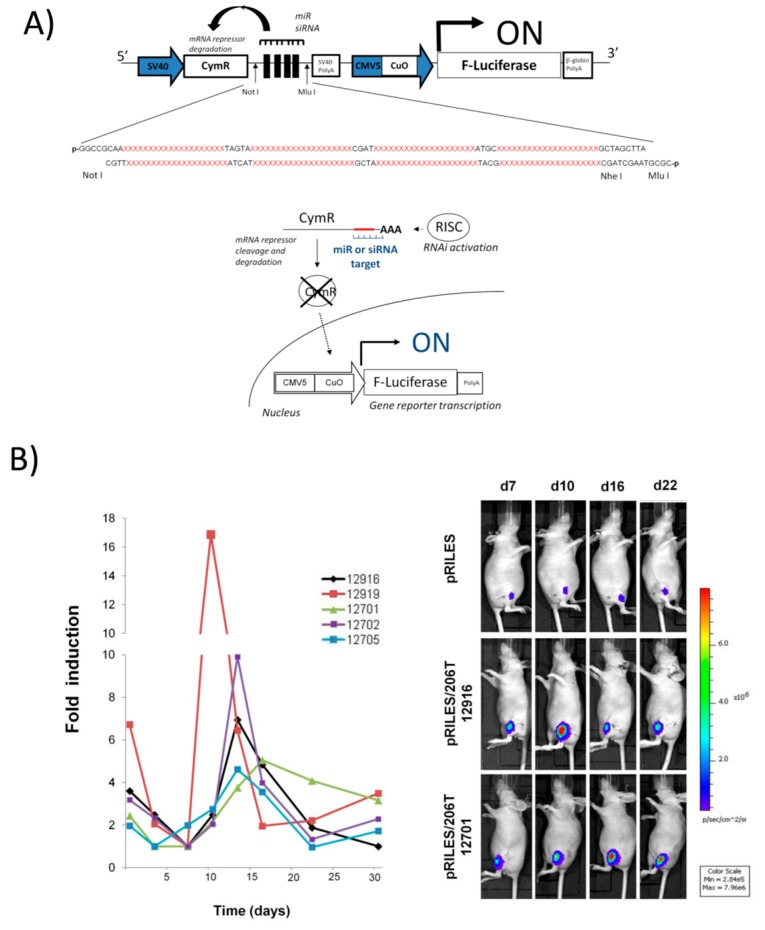
(**A**) Diagram representing the RILES monitoring method. When present in cells, target miRNA binds to the four complementary-block sequences located in the 3'-UTR of the CymR repressor transcript and activates the RNAi silencing complex (RISC) machinery. The CymR mRNA is then cleaved and degraded, resulting in loss of repressor production. The luciferase expression system is thus switched-ON, generating a positive bioluminescence signal; (**B**) Real time monitoring of miRNA-206 regulation during development of skeletal muscle atrophy. Two micrograms of pRILES/206T, designed to detect the expression of miRNA-206, were transfected in the tibialis anterior to transfect the skeletal muscles of nude mice. Three days later the left sciatic nerves were cut surgically to induce denervation and atrophy of the animals’ lower legs. Mice (*n* = 5) were thereafter scanned twice a week for the first three weeks and then once a week until day 35 (end point of our experiment). Left Kinetics of miRNA-206 expression detected in each individual mouse through bioluminescence imaging. Results are expressed as relative fold of luciferase induction by normalizing the bioluminescence values to the minimal value found before the bioluminescence peak for each mouse individually. *Right* Representative bioluminescence images collected from one representative mouse from the pRILES group and two representative mice from the pRILES/206T group at days 7 (d7), 13 (d13), 22 (d22) and 30 (d30). The number identifying each mouse during the longitudinal study is given. Reprinted from [85].

Recently, we [85] and other teams [86,87] developed an alternative approach to monitor positively the endogenous expression pattern of miRNAs in cells *in vitro* and *in vivo*. The systems are based on engineered regulatable expression systems, also known as genetic switches, such as the Tet-Krab, Tet R [88] and the Cumate [89] gene-switch systems. All of these genetic switch systems share the same characteristics. They comprise two expression cassettes, one encodes for a transcriptional regulator molecule which when expressed in cells binds to operator sequences located in a promoter region of a second expression cassette encoding for a transgene. The final decision to express or repress expression of the transgene is achieved by adding a chemical exogenous inducer, often an antibiotic such as doxycycline, tetracycline or cumate. Depending on the nature of the regulatable expression system used, the transgene expression can therefore be turned “ON” or “OFF” by adding the inducer in the culture media or in the drinking water of mice. We [85] reasoned that placing expression of the transcriptional regulator directly under the control of the endogenous RNAi machinery rather than an exogenous molecule would be an alternative way to turn-ON expression of the transgene. Consequently, if a reporter gene was used as a transgene, the system would generate optical signals reflecting the expression pattern of miRNAs. To this end, four complementary block sequences (miR T) of a miRNA of interest were sublconed in the 3'-UTR of the CymR transcriptional repressor gene [89]. In this way, when the miRNA of interest is present in cells, it binds to the miR T sequence and activates the RNAi silencing machinery. The CymR mRNA is then cleaved and degraded, resulting in lack of repressor protein production. The second expression cassette encoding for the luciferase gene is then switched-ON, generating a positive bioluminescence signal which can be monitored using standard bioluminescence equipment (Figure 3).

**Table 1:** Overview of the advantages and drawbacks of the current methods used to monitor expression of miRNAs. * Functional monitoring of miRNAs can be investigated by enriching and/or pulling-down specific partners of the miRNA biogenesis such as RLC for instance [38,39]. The same procedures can be applied to monitor the spatio-temporal resolution of miRNA expression in cells and in tissue samples. However, although these elegant procedures offer possibility to identify directly bound mRNAs to miRNAs in RLC complex, they are, not yet, standardized, require technical expertise and cannot be applied to the same samples during the course of longitudinal studies.

miRNA Monitoring Methods	Functional Monitoring	Spatio-Temporal Resolution	Advantages	Drawbacks	Ref.
Invasive Methods					
Northern-blot	No *	Difficult *	Qualitative, no amplification procedure, sensitive	Lysis, large amount of starting material, long procedure	[41,42]
Microarray	No *	Difficult *	High-throughput screening, qualitative, biomarkers	Lysis, amplification procedure, false hybridization, biased interpretations	[43,44]
Real-time PCR	No *	Difficult *	Qualitative, quantitative, fast and accessible procedure	Lysis, amplification procedure, false hybridization, biased interpretations	[46,47]
Deep sequencing	No *	Difficult *	High resolution, qualitative, quantitative, sensitive and specific	Lysis, long procedure, complex bioinformatic analysis	[49,50]
ISH	No *	Spatial	Spatial resolution, no amplification procedure, colorimetric method	Low sensitivity, long and complex procedure	[52,53]
Non-Invasive Methods					
Molecular beacons	No *	Yes	Positive monitoring, short fellow-up, fast procedure, theranostic probes	Low signal-to-noise ratio, low resolution	[60,62]
Negative read-out	Yes	Yes	Functional monitoring, qualitative and quantitative	Negative monitoring	[72,77]
Positive Read-out					
RILES	Yes	Yes	Positive monitoring, qualitative and quantitative, temporal resolution	Leakiness, immunogenicity, potentially interfering	[85]
TetKrab	Yes	Yes	Positive monitoring, qualitative and quantitative, temporal resolution	Leakiness, immunogenicity, potentially interfering	[86]
miR-ON	Yes	Yes	Positive monitoring, qualitative and quantitative, temporal resolution	Leakiness, immunogenicity, potentially interfering	[87]

Recently, we conducted a study validating the principle behind this novel positive miRNA monitoring system called RILES (RNAi-inducing Luciferase expression system). We demonstrated that RILES is a robust, sensitive and straightforward method for the non-invasive, real time monitoring of miRNA expression in cell lines and tissues of live anesthetised animals. Co-transfection of the HEK 293 cells with the RILES containing target sequences of miRNA-122 (RILES/122T) in the presence of increasing doses of synthetic miRNA-122 (miR mimic) resulted in induction of luciferase gene expression which was proportional to the dose of miRNA-122 mimic transfected in these cells. The calculated correlation coefficient *R^2^* value was 0.9321 demonstrating the linear range of detection of the RILES. The lower detection limit was determined in these assays as 0.05 nM which corresponds to the concentration of miRNA endogenously expressed in cells. The specificity of the RILES was further validated in control experiments performed with irrelevant synthetic miRNAs. No significant induction of luciferase expression was detected in cells transfected with the RILES/122T in the presence of synthetic miRNA-133 or miRNA-221. Similar experiments were performed with established cell lines to monitor the expression of endogenously expressed miRNA in these cells. Luciferase expression in hepatocarcinoma HUH7 and HLE cell lines transfected with either the RILES/122T or RILES/221T was found to follow closely the expression pattern of these two miRNAs detected by qRT-PCR. For example, when HUH7 cells were found to express the miRNA-122 with qRT-PCR, significant bioluminescence signals were detected in these cells transfected with the RILES/122T, but not when the cells were transfected with the RILES/221T to detect mRNA-221, not expressed in this cell type. 

Remarkably, the same trend of specificity and sensitivity was also found *in vivo* in small anesthetized animals. Using RILES, we successfully monitored the expression of miRNA-122 in the liver of mice and also the differential expression pattern of myomiRs-133, -1 and -206 expressed in the skeletal muscle of anterior tibialis of mice. Quantitative RT-PCR indicated that miRNA-206 and miRNA-133 were intermediately and strongly expressed respectively in the skeletal muscle of the mice. We also collected intermediate and elevated bioluminescence signals in the tibialis muscle of the mice transfected with RILES/206T or RILES/133T. In contrast, no significant bioluminescence signals were detected in a control group of mice transfected with pRILES/122T designed to detect liver specific miRNA-122, not expressed in muscle tissues.

Furthermore, we demonstrated that RILES has the potential to offer a temporal dimension analysis of miRNA expression which has never been obtained to date. Using an experimental model of muscular atrophy, we established for the first time the kinetic of miRNA-206 expression in five individual mice over a period of 35 days (Figure 3). While qRT-PCR indicated that the expression of miRNA-206 was elevated six days after the development of the atrophy and remained constant for the next 10 days, the data generated with the RILES was clearly different. The RILES indicated that expression of the miRNA-206 was not constant, but was in fact transient, characterized by three different expression peaks detected at day 13, 10 and 18 after development of the pathology. Moreover bioluminescence values detected from each mouse and plotted over time indicated that miRNA-206 expression was regulated over periods of time as short as four days and as long as 20 days. We also found that luciferase induction of miRNA-206 expression was heterogeneous between mice, ranging from a minimal value of two-fold to a maximum value of 17-fold of the basal level detected before development of the pathology. These data indicated that the expression of miRNA-206 is much more complex than that originally detected by qRT-PCR. The discrepancy between the two approaches is explained by the invasive nature of the qRT-PCR method which generates a bulk of information on miRNA expression produced from pools of several samples collected at different time points without taking into account either the temporal regulation of miRNA-206 or the individual’s miRNA-206 specificity in response to the muscular injury. Therefore, the trend of miRNA expression is better approached with the RILES which can be further exploited to improve understanding of the biological role played by the miRNA-206 in atrophied muscle tissues. Furthermore, information generated by the RILES could also be used as a rationale to develop new RNAi-based therapies. Indeed, underestimating miRNA-206 expression kinetic at the individual level might reduce the benefit of a miRNA-based therapeutic approach which aims at restoring (replacement therapy) or reducing (antagonist therapy) the expression of miRNA-206 for the treatment of muscular diseases [85,90,91].

However, the use of a miRNA reporter gene system such as RILES has some limitations. The long-term expression of a miRNA targeting sequence (miR T) in transfected cells might compete with the endogenous mRNA targets. As a result, RILES might interfere with the biological process studied. Other studies have addressed such issues [74,92,93] and demonstrated that a saturable effect of the miR T in cells is found only when the expression of transgenes bearing the miR T is driven by a strong promoter and when the number of miR T exceeds four. In our study [85], the weak SV40 promoter was used to drive the expression of the CymR transcript bearing the miR T and the number of miR T did not exceed four. Importantly, we did not find any statistical difference in terms of miRNA-206 expression between RILES-transfected tibialis muscles and non-transfected control muscles. This indicates that the fraction of miRNA-206 bound to the CymR repressor transcript was not compensated by overexpression of miRNA-206 and therefore suggests that RILES might be minimally interfering in cells.

In a similar way, Pichard *et al.* [86] have demonstrated that an engineered lentiviral vector, encoding for the tTR-KRAB repressor placed under the control of a miR T cassette, promotes efficient control of transgene expression in hepatocyte, macrophage and skeletal muscle-derived cells *in vitro* and *in vivo*. Indeed, when the GFP reporter gene was used as a transgene in the second inducible expression cassette, specific fluorescence signals were found in macrophages when the miR T sequence was designed to be complementary to the hematopoietic miRNA 142-3p, in hepatocytes when the miR T sequence was designed to be complementary to the liver specific miRNA-122, and in skeletal muscle when the miR T sequence was designed to be complementary to the muscle specific miRNA-133. In control cell lines which did not express the miRNA of interest or display a reverse miRNA pattern, no or only faint GFP expression was detected. The authors reported that a complete shut-off of the GFP expression in the absence of the miRNA of interest was found dependent on the cell type and on the promoter used to drive the TKrab expression. This, referred to as promoter leakiness, is a common drawback for most of the inducible expression systems described so far [85]. Similarly, in our study using RILES, we also found some basal expression of the luciferase gene in the absence of the miRNA of interest. However, this leakiness was at a low level and was not a major limitation for these two studies. However, as for other prokaryotic-inducible expression systems such as Tet R, Tet ON/OFF and tKRAB, their long term use in immunocompetent animal models is compromised by the immunogenic nature of the transcriptional repressors which are of bacterial origin [88,94]. This represents a serious limitation to the broad application of the miR-ON inducible reporter gene system in relevant immunocompetent animal models. Nevertheless, alternatives exist to overcome these problems. For instance, the mammalianization of the prokaryotic transcriptional repressor could be performed as it has been proved effective in other systems [95].

Despite these drawbacks and as stated by the authors [76,85,86], the use of regulatable miR-ON expression systems placed under the control of lineage-specific miRNA expression represent a robust method to achieve tight control of transgene expression in specific tissue types, with applications in gene therapy. Recently, Amendola *et al.* [87] have demonstrated that these miR-inducible ON systems could also be applied in replacement cell therapy. Engineered lentiviral vectors encoding for the tKRAB or the tTR repressor protein, placed under the control of hematopoietic lineage-specific miR T, were found to be very efficient in identifying and selecting *ex vivo* subsets of hematopoietic stem and progenitor cell populations. When transplanted into mice, these different isolated cell types were able to repopulate their respective spleen and bone marrow niches. Remarkably, expression of the reporter gene, here the GFP, within the *in vivo* niche was consistent with the pattern of miRNAs differentially expressed by the cells. This work is of importance as it provides evidence that programming the expression of a therapeutic transgene in a different type of stem cell might be possible according to the differential expression pattern of miRNA in progenitor cells, stem cells and differentiated and functionalized mature cells. Regarding the importance of stem cells in replacement therapy [96], this approach might guarantee tight control of transgene expression in mature cells once the stem cells have reached the injured organs and differentiated to regenerate the damaged tissues.

## 5. Concluding Remarks

Molecular imaging of miRNA is attracting increasing attention in the field of miRNA biology as it provides the unique opportunity to study the dynamic regulation of miRNA in a physio(patho)logical condition using animal models. The ideal system might be a probe which (1) minimally interferes with the cell biology; (2) enables the emission of positive optical signals when miRNA of interest is expressed; (3) is sufficiently sensitive to monitor expression of faintly expressed miRNA; (4) is compatible with the spatiotemporal study of miRNA expression in live organisms and (5) allows the monitoring of functionally active miRNAs, particularly those capable of repressing the expression of target mRNAs.

To date, molecular beacons have some potential for diagnostic applications, at least in *ex vivo* procedures, offering a rapid and effective method to screen for several miRNA tumour markers in human tissue sections. They also have a theranostic potential as reported in recent studies [63,65] but further confirmation is required in other animal tumour models. As stated in the previous sections, the main limitations of this approach are the low signal-to-noise ratio of fluorophores and the difficulty to deliver a sufficient amount of MB to tumour cells. The delivery of nucleic acids to targeted tissues *in vivo* remains a challenge, particularly when considering the intravenous administration route. Hence, molecular probes based on reporter genes seem more appropriate for *in vivo* monitoring of miRNA expression. Indeed, higher levels of molecular imaging probes can be produced in cells upon transfection. Furthermore, reporter genes can be subcloned into integrative vectors such as lentivirus expression systems for the longitudinal analysis of miRNA expression over periods varying from several days up to a month. It is worth noting that isotopic reporter genes, such as the sodium/iodide symporter (NIS), can substitute the luciferase reporter gene for use with SPECT/CT or TEP/CT nuclear medicine imaging scanners [97]. The expression of NIS has been used for more than 40 years in humans to diagnose and treat thyroid disease including cancer [98]. Therefore, miRNA imaging using the NIS as reporter gene might be feasible in the clinical context. Finally, other transgenes such as therapeutic cDNA, shRNA, miRNA antagonist and antagonist nucleic acid sequences can also be used instead of the reporter genes, opening up many applications in the field of gene and cell therapy.

In conclusion, each of the miRNA monitoring systems detailed in this review has its own advantages and drawbacks. At present, there is no one method capable of combining all the advantages of the different ways of monitoring miRNA and avoiding their limitations. Therefore, all of these approaches should be considered as complementary methods that ideally should be used in synergy to collect multiple and complementary biological information about miRNA expression and functions. This information will significantly advance our knowledge on biological processes involving miRNAs and will contribute to the development of more effective RNAi-based therapy.
